# Detection of Tephra Layers in Antarctic Sediment Cores with Hyperspectral Imaging

**DOI:** 10.1371/journal.pone.0146578

**Published:** 2016-01-27

**Authors:** Ismael F. Aymerich, Marc Oliva, Santiago Giralt, Julio Martín-Herrero

**Affiliations:** 1 AtlantTIC, University of Vigo, Vigo, Spain; 2 Centre for Geographical Studies (IGOT), Universidade de Lisboa, Lisboa, Portugal; 3 Institute of Earth Sciences Jaume Almera (CSIC), Barcelona, Spain; Glasgow University, UNITED KINGDOM

## Abstract

Tephrochronology uses recognizable volcanic ash layers (from airborne pyroclastic deposits, or tephras) in geological strata to set unique time references for paleoenvironmental events across wide geographic areas. This involves the detection of tephra layers which sometimes are not evident to the naked eye, including the so-called cryptotephras. Tests that are expensive, time-consuming, and/or destructive are often required. Destructive testing for tephra layers of cores from difficult regions, such as Antarctica, which are useful sources of other kinds of information beyond tephras, is always undesirable. Here we propose hyperspectral imaging of cores, Self-Organizing Map (SOM) clustering of the preprocessed spectral signatures, and spatial analysis of the classified images as a convenient, fast, non-destructive method for tephra detection. We test the method in five sediment cores from three Antarctic lakes, and show its potential for detection of tephras and cryptotephras.

## Introduction

Since Thorarinsson established the fundamental principles of tephrochronology in the 1940s [1], volcanic ash layers of pyroclastic airborne material, known as tephras, are routinely used as time markers for synchronization of paleoenvironmental events across wide geographical regions. While studying the historic eruptions of the Hekla volcano in Iceland, he observed that individual tephra layers could be correlated over large areas [2]. The reason behind the use of these volcanic ash layers as time markers is that the ash from explosive eruptions covers vast surfaces and therefore affects vast areas almost instantaneously on a geological time scale. Furthermore, the chemical composition and microstructure of the ash allows the identification of different pyroclastic events. Where tephras can be detected and recognized, they give us significant information for correlating and dating past geological, environmental, climatic, and archaeological events. The detection of tephras is thus increasingly important in geological sciences and other disciplines. It provides, for instance, information about the history of volcanic activity and eruptive styles by establishing time-space relations between volcanic events [3]. The thickness and distribution of tephras allows, among other information, the estimation of magma volumes [4], the study of different types of pyroclastic deposits [5, 6] and provides an estimation of the frequency of the eruptions [7].

Tephras present in cores extracted from locations close to the originating volcanic eruption may be several millimeters to centimeters thick [8], and many can be distinguished by the trained eye of the expert. But significantly fewer ash particles are deposited as the study site is located further away from the volcanic eruption, or when the wind patterns negatively affect the ash dispersion in the area, and then we speak of cryptotephra, “hidden tephra”, indistinguishable to the naked eye, up to the point that eruptions happening at 1 500 km, for instance, can leave just a thin, non-visible tephra layer [9]. The detection of tephras in cores can be performed by means of magnetic, chemical or spectroscopic analysis, X-ray diffraction, and small volcanic shard counting, among others [10, 11]. These methods are often costly and time consuming, and may require partial or complete destruction of the sediment sample, seriously hindering or even preventing any further use or studies of the samples. This is especially negative when the samples come from regions involving special difficulties regarding access, fieldwork conditions, extraction permits, protection measures and environmental fragility, such as Antarctica.

In 1998, Caseldine [12] recognized the importance of finding a quick and non-destructive technique for tephra detection, and showed how a combination of reflectance and luminescence could identify distal tephras. In 2008, Gehrels et al. [13] reviewed several conventional methods commonly used for cryptotephra detection and presented some results with non-destructive and rapid techniques on peat cores with known cryptotephrastratigraphy, such as X-ray fluorescence spectroscopy, magnetic susceptibility, and reflectance (non-imaging) spectroscopy. They concluded that these non-destructive methods had difficulties to detect cryptotephras but might be interesting for the detection of thin layers or dispersed, macroscopic non-visible tephra material. In the specific case of spectrophotometry, they stated the importance of a flat surface for effective measurements and the reduced efficacy when applied to sediments where other sources of minerogenic layers are likely to occur.

Closely related to conventional spectroscopy, hyperspectral imaging, also known as imaging spectroscopy, allowing simultaneous spectroscopic measurement of every pixel in an image, has been applied to a wide range of fields [14–22] during the last decades. Among them, geology has not remained oblivious to hyperspectral imaging, and indeed it was one of the main drivers of some of the pioneer devices [23]. In 2013, Chen et al. [24] determined chlorophyll-a concentration in sediment profiles from its visible reflectance signature using a laboratory spectrophotometer. They found a trough in the spectral signature at 650–700 nm, which served as marker for chlorophyll-a concentration, useful to discriminate organic matter. Very recently, Grosjean et al. [25] resorted to a hyperspectral scanner for the same purpose, using the relative absorption band depth at 660 and 670 nm in what could be considered a multi-point extension of Chen’s work, and suggested that advanced statistical techniques could provide interesting information about other substances and minerals. Here we propose to apply pattern recognition techniques to hyperspectral images as a feasible technique for rapid, cost-effective and non-destructive detection of tephras.

### Hyperspectral imaging

After a long time hindered by the high cost and operational complexity of the available hardware, in recent years hyperspectral sensors have become available at reasonable cost and practicality with the introduction of new generations of compact monochromators that can be combined with standard imaging sensors and computing equipment, taking advantage also of high throughput camera interface standards developed and mass-produced for industrial machine vision. All this has finally made possible for hyperspectral technology to get out of just a few heavily funded laboratories, such that its full potential can be used for many applications with reasonable costs and operational complexity [26, 27].

The term hyperspectral refers to the capability of these imaging devices to measure the intensity of incoming light at many different wavelengths, compared to classical multispectral imagers, where only a few spectral bands (a few tens or dozens, at most) are output. However, the difference is not just the number of bands but the fact that the hundreds of spectral bands of a hyperspectral system are contiguous and very narrow, sampling the portion of the spectrum covered by the device (which differs from device to device) in what could be considered the spectroscopic equivalent of Nyquist conditions for digital sampling [28]. In this sense, hyperspectral imagers allow the reconstruction of the spectral signature of a target, quite similarly to field or lab spectrophotometers, while multispectral imagers only provide a few discrete measurements of integrated, relatively wide, disjunct regions of the spectrum.

In this respect, it is important to stress the difference between hyperspectral imaging (or imaging spectroscopy) and non-imaging or conventional reflectance spectroscopy. In fact, some degree of confusion seems to exist even in specialized literature, where spectral measurements obtained with a field spectrometer are sometimes referred to as hyperspectral, most likely because field spectrometry can be used as a source of ground truth to reference hyperspectral images, or to assess the potential of hyperspectral imaging for a given application [29].

In general, reflectance spectroscopy tries to obtain information about the nature, structure and state of a target, and specially about its surface or a small depth just under its surface, depending on the material, by measuring its interaction with light. When light from a measurable source, also known as incident radiation or downwelling, reaches a target, the impinging photons interact with it, according to their energy and therefore to their wavelength (shorter wavelength photons are more energetic): Some are absorbed and transformed into heat, some are absorbed and released sooner or later, perhaps at a new wavelength, some are transmitted, by passing unaltered through the target, and some are backscattered or reflected. The reflected photons constitute what is called the reflected radiation or upwelling. By measuring and comparing the downwelling and upwelling, the reflectance spectrum of the target can be computed, as a measure of the proportion of photons reflected at each wavelength, which depends on the nature, structure and state of the target surface and right under it. The reflectance spectrum therefore acts as a kind of spectral signature which may help us identify the nature and state of a target. In fact, our eyes do that all the time: They act as a three-band spectrophotometer to produce the visual sensation we call *colour* (the perceptual equivalent of a low-resolution reflectance spectrum), which our brain uses to distinguish objects and qualities alongside other high level attributes such as shape or size.

In conventional reflectance spectroscopy, a monochromator splits the light entering through an optical fiber and directs each wavelength (according to the nominal spectral resolution of the device) to a different region of a photodiode array or linear imaging device, usually a linear CCD or CMOS, composed of a single row of sensitive cells. Thus, a spectrometer measures light coming through a single fiber (or channel), providing a spectral signature for the entire area integrated by the aperture of the fiber. Dual-channel (and higher) spectrometers exist, which are in fact two or more stacked single-channel spectrometers, allowing, for instance, the measurement of the downwelling or incident light at the same time as the upwelling or reflected light.

A hyperspectral sensor, or imaging spectrometer, also has a monochromator that splits light into different wavelengths. However, the light entering the monochromator is not entering through a single fiber but through an imaging lens very much like those used for standard cameras, usually with special coatings to cope with the extended spectral range of the system. Hence, the monochromator does not split a single beam of light into a single linear detector array. The monochromator has to deal at the same time with a high number of different pixels, whose light splits and diverts to different rows of a focal plane array detector (usually a CCD or CMOS matrix), thus getting at each detector row the spectral signature of each pixel.

Pushbroom hyperspectral sensors deal with one image line at a time, by replicating it onto the matricial detector, where “copies” of the line are replicated on the different rows of the detector, but at different wavelengths for different rows along what is called the *spectral* axis of the detector, in contrast to the *spatial* axis, along which the different pixels of the image line are to be found. Pushbroom image sensors, therefore, acquire images by scanning the target line by line [30]. Snapshot hyperspectral sensors are now being developed, still with relatively poor spectral to spatial resolution trade-offs, but allowing simultaneous imaging of a scene, instead of having to build it line by line [31].

The main difference between conventional reflectance spectroscopy and imaging spectroscopy or hyperspectral imaging is thus the fact that hyperspectral imaging provides *images*, i.e. sets of adjacent, spatially-related, and geometrically or geographically referenced pixels for which individual reflectance spectral signatures are provided, while a field or lab spectrometer provides “just” a single reflectance spectral signature (or a few) for a sample as a whole. To obtain with a field spectrometer a dataset equivalent to a hyperspectral image, many individual measurements would have to be performed sequentially, each time pointing the optical fiber to a slightly different part of the target by displacing one with respect to the other along the imaging axes, namely a displacement equal to the corresponding aperture of the fiber, which would be required to be much smaller than usual. As typical hyperspectral images have several millions of pixels, several million measurements and displacements would have to be performed with a spectrometer, which shows the impracticality of such an approach, not to mention the technical difficulties of such an arrangement, and the need of specific monochromators able to handle the spatial diversity inherent to imaging.

We propose a step forward from non-imaging reflectance spectroscopy towards fast non-destructive detection of tephras and cryptotephras by means of hyperspectral imaging of sediment cores and subsequent analysis of the images using machine learning techniques. We tested this methodology in five cores extracted from the bottom of three lakes in Livingston Island (Antarctica).

## Methods

### Study area and sediment cores

**Fig 1:**
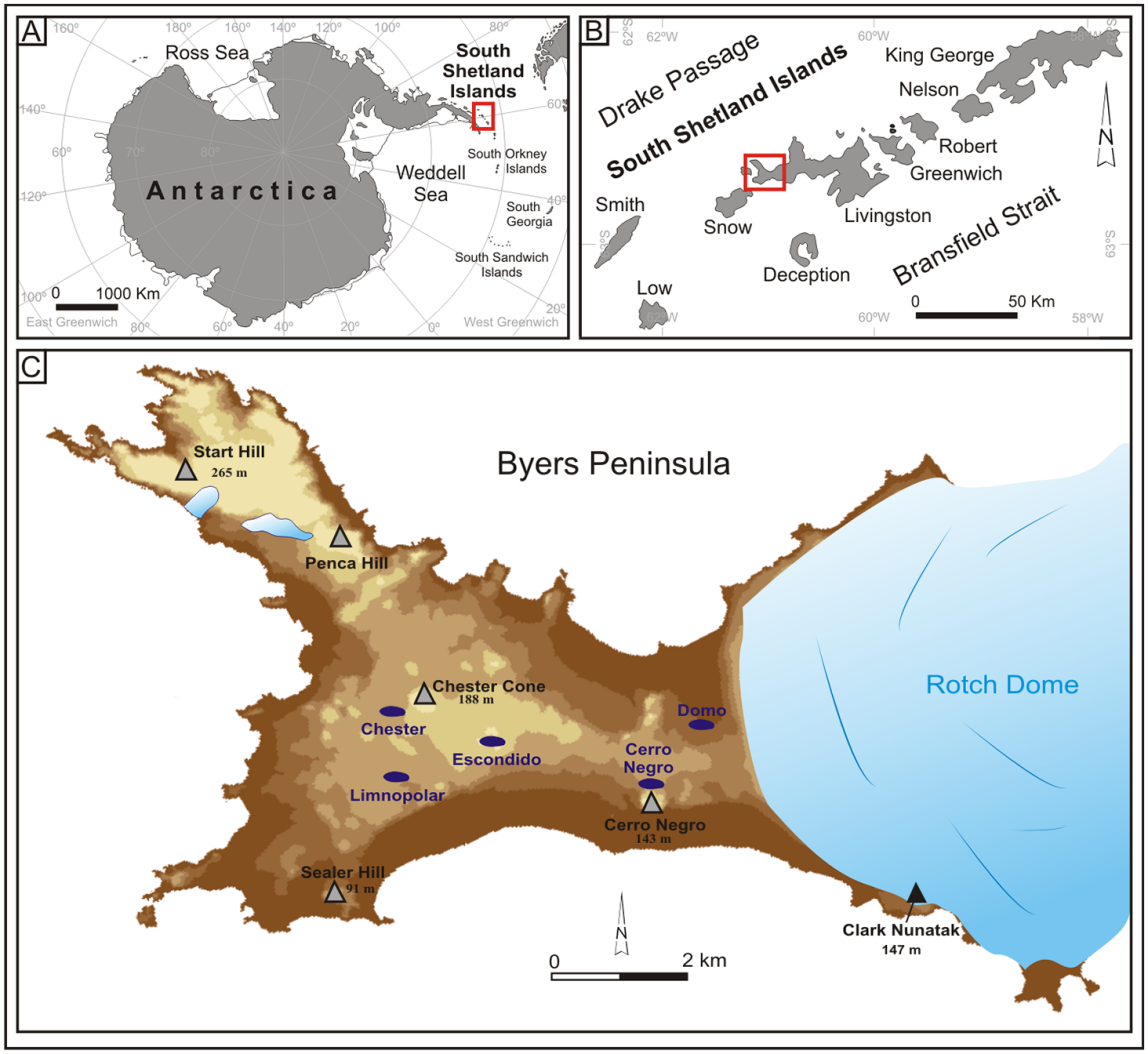
Location of Antarctic lakes sampled for this work. A) The South Shetlands within the Antarctic continent; B) Byers Peninsula within the South Shetlands; C) Target lakes in Byers.

We used five lake sediment cores retrieved from Byers Peninsula, an ice-free area at the westernmost tip of Livingston Island, the second largest of the South Shetlands archipelago (Antarctica) after King George Island. All research activities were conducted following the regime of special environmental protection of Byers Peninsula (Antarctic Specially Protected Area No. 126) which is sustained under the Antarctic Treaty System. The corresponding permit was issued by the Brazilian Antarctic Program (PROANTAR). This peninsula extends over latitudes 62°34’ S—62°41’ S and longitudes 60°54’ W—61°12’ W. Byers Peninsula constitutes the largest ice-free environment in the entire archipelago, with a surface of ca. 60 km^2^, see Fig 1. Its relief is structured by a relatively flat central plateau (40–100m asl), surrounded by Holocene marine terraces (2–15 m). The highest elevations are several volcanic plugs, such as Start Hill at 265m, Chester Cone at 188m, and Cerro Negro at 143m.

The lithology of Byers Peninsula is composed of a succession of marine and volcanic Upper Jurassic to Lower Cretaceous sediments overlaid by Quaternary lacustrine and volcaniclastic deposits [32, 33]. The retreat of the Rotch Dome glacier during the last millennia has been accompanied by an extension of periglacial processes in the recently deglaciated areas. Another consequence of the glacier shrinking is the formation of tens of lakes and ponds in the depressions carved by glacial erosion in the central plateau. Glacial and periglacial processes and landforms have played a prominent role in present and past sediment transport dynamics within the lake catchments. Among these lakes we find Limnopolar, Escondido, and Cerro Negro, all covered by ice during 9–10 months per year.

The most active volcano in the Antarctic Peninsula region [7] is located in Deception Island, 45 km south of Byers Peninsula. The origin and geological evolution of Deception Island have received renewed attention over the last years [34], together with its recent eruptive dynamics [35, 36]. Some of the volcanic eruptions of Deception Island have deposited ash particles over many areas of the western Antarctic Peninsula, as inferred from the thick tephra layers present in the glacier fronts in Livingston Island [37] as well as in many lake sediment records from several ice-free environments in the South Shetlands [38–42]. Complete sedimentary sequences of three lakes were retrieved in 2008 (Limnopolar) and 2012 (Escondido and Cerro Negro), see Table 1. The coring operations were conducted in the early austral summer season so that the ice cover of the lakes was used as drilling platform. An UWITEC gravity corer was used to collect the most recent sediments, whilst a 90 mm UWITEC piston corer was used to retrieve the deeper sediments. The sediment cores were sealed in the field using specific plastic caps and tape and stored at a constant temperature of +4°C. Once in the laboratory, the cores were split longitudinally using two diamond saws for the plastic liner and a thin steel wire to section the sediments into two symmetric halves. The main lithostratigraphical properties were described following the standard procedure. A detailed chemical and morphological characterization of the tephra in the sediment cores used in this work can be found in Liu et al. [36].

**Table 1:** Antarctic lake sediment cores used in this work.

Core	Lake	Depth	Lat/Lon	Altitude
ES12-0302 ES12-0301	Escondido	5.2 m	62°37’06.57”S 61°03’36.50”W	92 m
CN12-0301G	Cerro Negro	2.2 m	62°37’41.30”S 61°00’19.99”W	100 m
LIM08-A2A LIM08-F1A	Limnopolar	4.5 m	62°37’23.65”S 61°06’23.67”W	65 m

### Hyperspectral image acquisition

We acquired hyperspectral images from the sediment cores at the Sedimentary Geology and Global Change laboratory of the Institute of Earth Sciences Jaume Almera (ICTJA-CSIC) using a custom pushbroom hyperspectral imager. The imager uses a Hyperspec VNIR monochromator from Headwall Photonics, with a Schneider Kreutznach broadband-coated Cinegon 1.4/ 4.8 mm lens, and a high quantum efficiency, low noise, global shutter EV76C661 CMOS detector from e2v Semiconductors as focal plane array. The spatial axis has 1280 pixels, and 368 spectral bands are obtained from the 1024-row spectral axis, covering the 400–1300 nm range with 10-bit radiometric resolution. The system is interfaced to a laptop through a Gigabit Ethernet interface using the high speed GigE Vision standard. Scanning at 30 frames per second, the throughput is 141.3 Mb/s, which goes up to 471.4 Mb/s at 100 frames per second. The huge amount of data produced by hyperspectral systems has been one of the bottlenecks explaining the high costs of the hardware of the earlier systems. However, nowadays, by using direct memory access (DMA) and network cards able to process Gigabit Ethernet network traffic with very low CPU usage, standard PCs are able to acquire, store and visualize the images in realtime during acquisition, to the extreme that, with the aid of a consumer multicore GPU graphics card, they even can georeference images in realtime during aerial acquisition [30]. The program to control, perform, preprocess and display the acquisition was entirely programmed in C and C++ by the authors.

**Fig 2:**
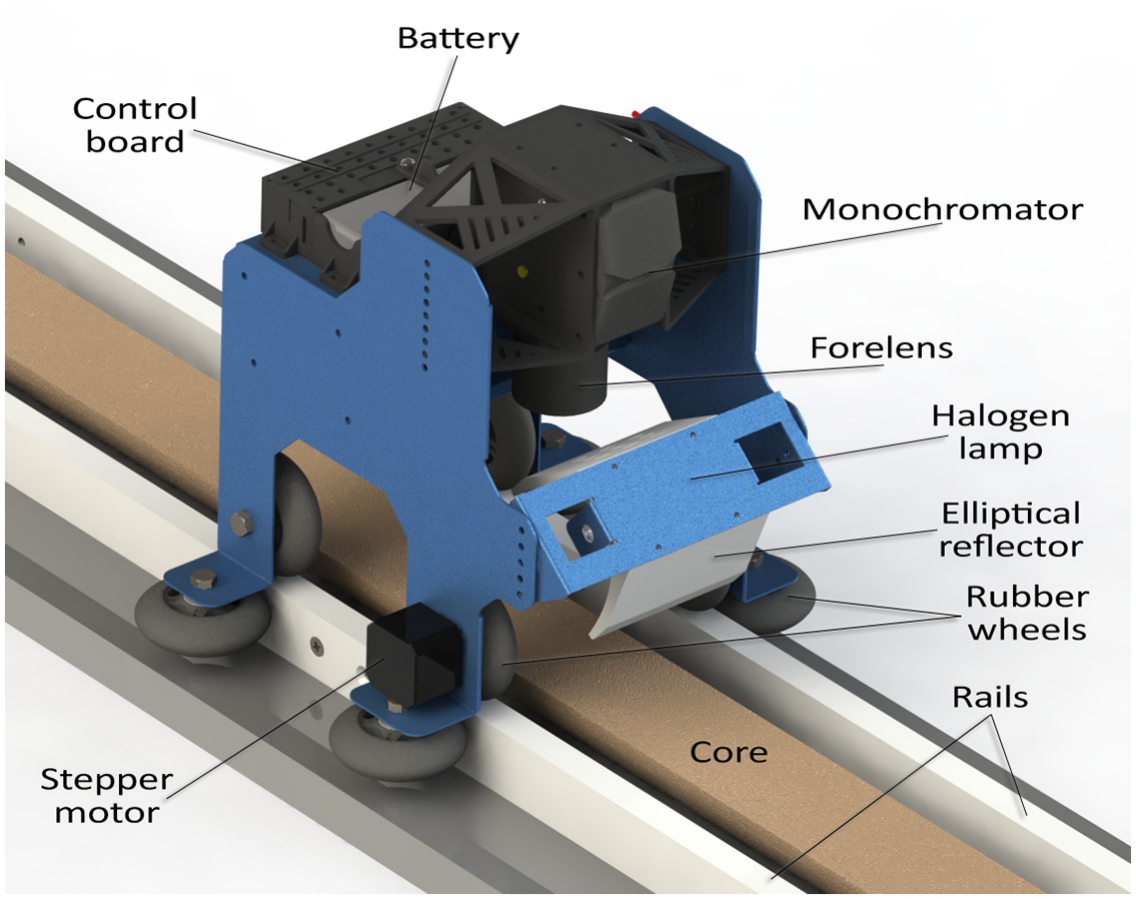
Hyperspectral camera dolly. Camera dolly on rails moved by a microstep-driven stepper motor and carrying the hyperspectral camera and a halogen light source within an elliptical reflector, astride a sediment core.

For scanning the cores, we designed and built a small dolly on rails moved by a 200-step stepper motor with a ×8 microstepping driver controlled by the same computer performing the image acquisition, via an Atmel ATmega32u4 microcontroller interfaced by USB. The whole system is powered by a rechargeable 4S 3 300 mAh LiPo battery providing well over one hour of operation on a single charge. The dolly guarantees a smooth travel at constant speed, and constant distance from the lens to the core surface, with the rails at both sides of the core, see Fig 2. It also carries two halogen lamps inside a custom elliptical reflector designed to provide a uniform bright stripe of illumination across the core, right under the camera lens. We set the speed of the dolly for 0.5 mm along-track spatial resolution, whereas the geometry of the system (focal length and distance to target surface) provided an across-track spatial resolution of 75 μm. At this resolution, 1.4 GByte of image data are produced per scanned meter.

### Hyperspectral normalization

It is a well known fact of reflectance spectroscopy that the angle of incident radiation and the angle of observation of the reflected radiation is an important source of variation of the observed reflected light. The bidirectional reflectance distribution function (BRDF [43]) defines how the reflected light varies with the angle of incidence and the angle of observation. Several standard models can be assumed for a given type of material and surface texture to discount the BRDF effect when the geometry of the surface, and therefore the incidence and observation angles, are known, but when they are not known, the BRDF introduces a notable source of uncertainty in the measurement of the reflectance spectrum. The microtopography of the surface of the sediment core scanned with the hyperspectral imager is a complex variable which we do not measure, but cannot be assumed to be ideally flat. It could be measured, for instance by triangulation with a laser line, but the very high spatial resolution of the hyperspectral scan would convey problems of registration accuracy, introducing an additional source of uncertainty.

We chose to deal with the microtopography of the samples by means of hyperspectral normalization [44], which also tackles the effect of shadows. Shadows occur when a topographic accident, such as the edge of a crevice or a raised clump, sheds its shadow on adjacent areas of the surface, by interposing itself between the light source and part of the surface being scanned. Hyperspectral normalization is provided by the following expression,

$${\hat{S}}_{i}=\frac{S_{i}-\min_{j}\left\{S_{j}\right\}}{\sum_{k}^{N}S_{k}-N\min_{j}\left\{S_{j}\right\}}$$ (1)

where ${\hat{S}}_{i}$ is the normalized reflectance spectrum at band *i*, *S*_*i*_ the original value, and *N* the total number of bands. A detailed justification of Eq (1) and how it copes with varying incident angles and shadows is out of the scope of this paper, but the interested reader can find it in [45].

### Detection of tephra suspect pixels

Our objective was the detection of tephra layers in the hyperspectral images of the sediment cores. For this purpose, as a first step we resorted to supervised per-pixel binary classification (“tephra” and “non-tephra” classes) of the images, by means of Self-Organizing Map (SOM) clustering of a training set of tephra and non-tephra pixel samples. Clustering is a statistical (and therefore also an algebraic and thus also a geometrical) analysis that groups a set of elements into classes or clusters according to a certain measure of similarity or similarity metric, such that elements belonging to the same cluster are more similar to each other (as per the selected metric) than elements in different clusters. The specific clustering method and the specific similarity metric determine the clusters for a given dataset, and different metrics, methods and even runs of a given method (when stochastic) may produce different clusters for the same dataset.

#### Self-Organizing Maps

SOMs [46–50] are a type of artificial neural network that map highly dimensional feature spaces into lower dimensional manifolds. A SOM consists of a set of neurons, called nodes, usually organized on a regular low-dimensional grid. Each node is identified by its coordinates in the grid, and has an associated vector in the input feature space, called prototype. The prototype vectors of a trained SOM are such that the map will preserve the topology of the input feature dataset. This implies that prototype vectors that are neighbors in the input feature space correspond to nodes that are neighbors in the SOM grid. This is achieved by means of an unsupervised competitive learning algorithm. The term unsupervised refers to the fact that the training samples are not labeled as belonging to different classes: for the SOM at the learning stage, all samples are equal and they are not put into different classes. The term competitive refers to the fact that nodes compete for which is the one that best represents the current sample during the learning process. The nodes are initialized with random prototype vectors, and then a training sample is selected from the input dataset. The node with the prototype vector closest to the sample, according to the metric of choice, is selected as the winner or best matching unit (BMU), and its prototype vector is updated by moving it closer to the sample. The nodes in the neighborhood of the BMU are also updated in the same direction, according to a tunable neighborhood function, usually a Gaussian centered in the BMU. In this way the excitation of a node influences its neighborhood, a far resemblance to a usual trait of biological neuronal systems. Then another training sample is selected and the process repeated. This goes on until some stopping criterion is reached, e.g. when the variation of the prototype vectors is small enough.

The usual update expression at iteration *i* for the prototype vector **w**_*C*_ associated to the node located at point *C* of the map is

$$w_{C}^{i+1}=w_{C}^{i}+\eta^{i}h^{i}(∥C-C_{\text{BMU}}∥)\left(x^{i}-w_{C}^{i}\right)$$ (2)

where *η*^*i*^ is the learning rate, *h*^*i*^(*r*) is the neighborhood function, *C*_*BMU*_ is the location of the BMU, and **x^i^** is the input data vector or sample presented to the SOM at iteration *i*. The neighborhood function *h*(*r*) defines the region of influence of the nodes, usually a Gaussian centered at *C*_*BMU*_ (*r* = 0), with tunable variance specifying the spread of the neighborhood. At each learning step, all neurons within the neighborhood defined by *h*^*i*^(*r*) are updated, and all the rest kept constant. The learning rate and the neighborhood size decrease with time as the learning progresses. This implies that at first the nodes evolve quickly and large variations are allowed, but later a finer tuning gets smoother results.

#### U-Matrix

**Fig 3:**
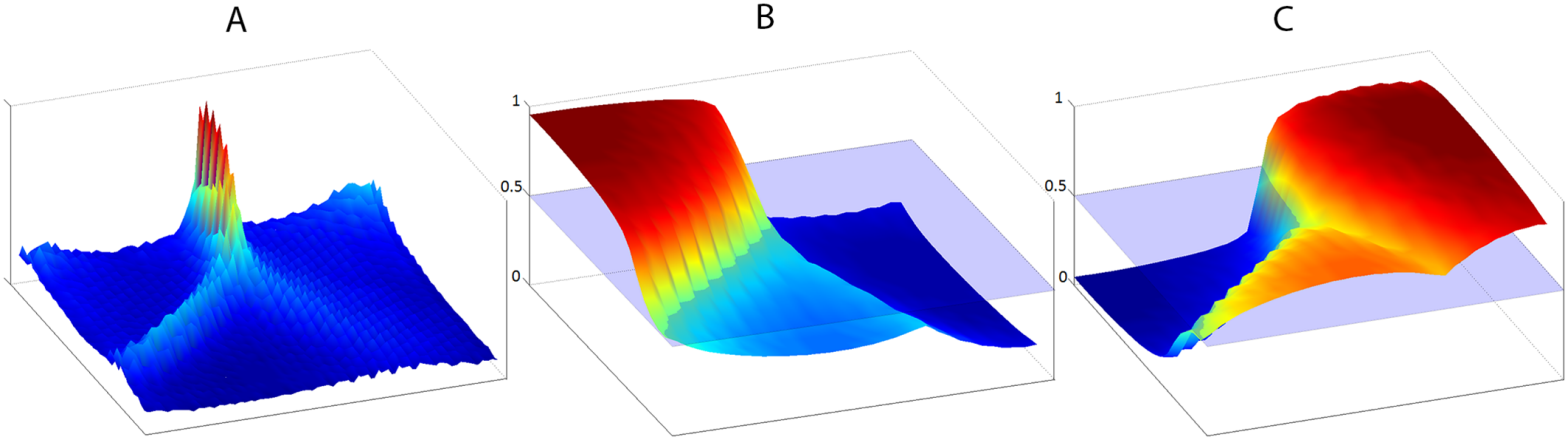
U-matrix and confidence matrices for the “tephra” and “non-tephra” classes. These are different ways to visualize the behaviour of a trained self-organizing map. A: U-matrix, where hotter colours indicate bigger distances in feature space (hyperspectral space) between nodes adjacent in the map (the scale is not relevant, it is the relative differences in different regions of the map that are significant, with warm and hot ridges indicating decision boundaries between clusters in feature space); B: Confidence matrix of “tephra” class; C: Confidence matrix of “non-tephra” class. Confidence matrices reflect a measure of confidence from 0 (null) to 1 (total confidence) that a hyperspectral signature mapped to a given node of the SOM belongs to the corresponding class. The horizontal plane marks the 0.5 level, i.e. the unbiased decision threshold.

By way of the above process, while the nodes remain static in their regular grid in SOM space, usually a two-dimensional square or hexagonal grid, their associated prototype vectors are rearranged in input space to form a lower-dimensional manifold within that space, which is expected to provide an accurate description or fit of the input dataset used for training [51, 52]. This topology-preserving reduction of dimensionality is what makes feasible to see in output space potential structures and relations hidden in the high-dimensional input space. One way to visualize this is through the unified distance matrix or U-matrix [53], a representation of the distances among the prototype vectors of adjacent nodes. For a *P* × *Q* hexagonal map, the U-matrix has (2*P* − 1) × (2*Q* − 1) elements, made of *P* × *Q* elements corresponding to the map nodes, surrounded by six elements that contain the distance to each of the six adjacent nodes. The nodal elements contain the average of the surrounding elements, i.e. the average of the distances to all the adjacent nodes. The conceptual construction of the U-matrix is as follows: 1) Take the original *P* × *Q* hexagonal grid of the map and insert room for an additional hexagonal cell in between every pair of original adjacent cells. 2) Fill in these new cells with the distance between the prototype vectors of the corresponding nodes. 3) Finally, fill in the original cells with the average of all the adjacent new cells. This (2*P* − 1) × (2*Q* − 1) hexagonal grid is the U-matrix. The U-matrix is usually represented in a color scale with hot/cold colors indicating bigger/smaller internodal distances. Clusters can then be visually identified as cold or flat regions separated by warm or hot walls, see the leftmost graph in Fig 3.

#### Confidence matrices

Note that up to this point no information about the different classes of the training samples has been used. We can incorporate this information through the so-called hit or response matrices. A hit matrix is yet another representation of the SOM where each node is assigned the number of times it is the winner node or BMU when a labeled subset of the training set is presented to the SOM. All samples belonging to a class are sequentially presented to the map, and for each sample the BMU is found and its hit counter increased. This produces the hit matrix for that class, and the process is repeated for each class. A higher number of hits, and therefore hotter regions, will appear in the hit matrix in the areas of the map that can be identified with the class. A good coherence between the U-matrix and the hit matrices, with the “hot walls” in the U-matrix showing a good correspondence with the boundaries between the hotter areas of the different hit matrices, will indicate that the input features (in our case the normalized spectral signature) and the mapping performed by the SOM fit well the problem of discriminating between those classes (again note that the SOM and the U-matrix are obtained without any reference whatsoever to the classes, only used to build the hit matrices). Hit matrices, however, tend to not behave well, because two situations may arise that they do not properly reflect: 1) Several nodes in the map may have responses to a given sample very close to that of the BMU, but only the winner node or BMU (even if it is just by an infinitesimal advantage) gets its hit counter increased, i.e. it takes all the merit rather undeservedly. 2) Both, very high responses (very good matches between the sample and the winner prototype) and very low responses (very bad matches; even if still the highest in the whole map for that specific sample, e.g. outlier samples too far from the mapped manifold) have equal impact on the hit matrix: one hit. To tackle this, a regularized version of the hit matrices can be built by accumulating the overall response of the map to each sample, instead of just one hit being assigned to the BMU. This can be achieved by using the fuzzy response function proposed in [54],

$$g\left(x,C\right)=\frac{1}{1+(∥x-w_{C}∥/\bar{\epsilon })}$$ (3)

where **x** is the sample, *C* is any node location in the map, **w**_*C*_ its corresponding prototype vector, and $\bar{\epsilon }$ the average distance of all training samples to their corresponding BMUs. For each sample in the class, *g*(**x**, *C*) is computed for every node *C* in the map and accumulated into the corresponding element *C* of the response matrix.

The response matrices can then be used to obtain confidence matrices, see Fig 3. Confidence matrices are computed by normalizing each response matrix by the total sum of all the response matrices. Thus, each node can be assigned values of confidence in membership of each class, whose sum adds up to 1 (meaning that we have total confidence in the node belonging to any of the classes). As we deal here only with two classes, tephra and non-tephra, all nodes with a tephra-class confidence greater than 0.5 (the horizontal plane in Fig 3) can be considered tephra suspects, but more generally a tunable threshold can be used to allow finely tuned classification results, for instance to accommodate a circumstantial need for a higher sensitivity, and therefore a bigger amount of false positives, or the other way around, less sensitivity to greatly limit false detections at the expense of risking missing the faintest cryptotephras.

By setting this threshold, clusters are finally automatically defined in the map, by the union of all nodes having confidence levels greater than the threshold for the given class. Classification of any pixel can then be performed by finding the corresponding BMU in the SOM, and then comparing its tephra-confidence level with the threshold, i.e. checking to what cluster belongs its BMU.

#### Classification

The process to achieve per-pixel classification of the hyperspectral image can be summarized as follows:

1. A training image is selected, with tephra and non-tephra sections clearly identified.
2. The spectral signatures of all pixels in the image are normalized as per Eq (1).
3. A representative set of sample pixels is selected, trying to include all varieties present in the core. Their normalized spectral signatures are the feature vectors that will be used as inputs to the SOM. The pixels are assigned a label according to their class (tephra or non-tephra). The set is divided into two sets: a training set and a validation set, both containing samples of both classes.
4. Suitable parameters are selected for the SOM (size, learning rate, neighborhood function), and the SOM is trained with the training set, irrespective of the classes.
5. The U-matrix and the confidence matrices are computed and then contrasted. If they do not show a good degree of coherence for the discrimination of tephra and non-tephra samples, the SOM is reparameterized and trained again.
6. A confidence threshold set on the tephra confidence matrix is used to classify the validation set into tephra and non-tephra samples. Samples are classified by computing their BMUs and testing their corresponding tephra confidence level against the threshold of choice. Then the overall accuracy and observer agreement index, OA and *κ*, are computed to estimate the goodness of the classification [55]. If they are not satisfactory, the SOM is reparameterized and trained again.
7. The SOM is ready for automatic detection of highly confident tephra-suspect pixels in the whole image used for training, or in images of other cores with similar characteristics.

### Detection of tephra layers

By applying a threshold to the tephra confidence matrix, individual tephra-suspect pixels are detected. But false positives arise due to classification errors, when non-tephra pixels are classified as tephra-suspects, because the overall accuracy will never be 100%, but also due to scattered volcanic particles not belonging to tephra layers. These scattered particles may be the result of accidental dragging during the longitudinal split of the core in two halves, or they may be reworked volcanic material previously deposited in the catchment, later incorporated to the lacustrine sediments by run-off or other sedimentary transport mechanisms.

To avoid the influence of this scattered volcanic material, we take advantage of the fact that our targets, tephra layers, have a definite spatial arrangement: In laminated sediments tephras come in layers, and therefore we should be interested only in tephra-suspect pixels that belong to loosely horizontal stratified aggregates.

Therefore, firstly we get rid of any tephra-suspect pixels that do not form substantial spatial aggregates, by means of the standard mathematical morphology operators erosion and dilation, with a suitably-sized structural element, smaller than the smallest aggregate considered significant [56]. These operations are performed on the binary mask resulting of binary classification of the whole image into tephra and non-tephra suspects.

Then, we horizontally project the result of the morphologically filtered per-pixel detection across the sediment section, to obtain a tephra profile of the sediment core where peaks can be assumed to have a high probability of being tephra layers. We then look for peaks in this profile, and use the ratio between their height and width as an indicator of their spatial coherence as a layer.

Variable thresholds allow the user to consider different scenarios for the specific particulate found. For this purpose, we developed an interactive graphical user interface where the expert can change the parameters and see the impact on the detection results in real time, having complete control over the whole process.

### Validation

**Fig 4:**
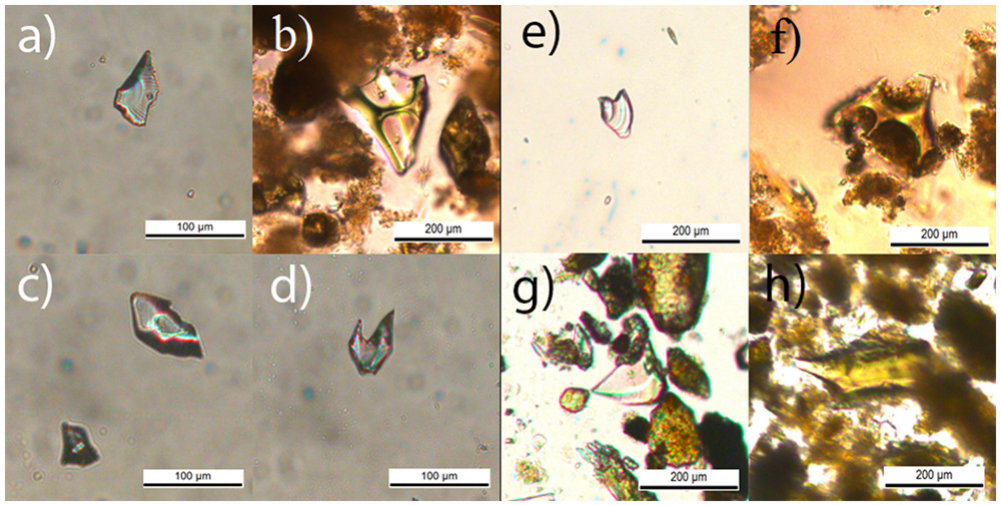
Shards found in suspect cryptotephras analyzed under the microscope. Shards from cryptotephras: a, b) ES01-CT1 (29.5 cm); c, d) ES01-CT2 (43 cm); e, f) ES02-CT1 (25 cm); g, h) ES02-CT2 (36 cm).

We needed some mechanism to assess the reality of the detections provided by the proposed method as a means of validating its efficacy. This requires the expertise of seasoned tephrochronologists. Substantial volcanic deposits easily visible in the core are readily identified by the experts using the naked eye. However, some cores also have other more subtle volcanic layers that cannot be easily identified by eye. A reliable, but costly because staff-intensive, method to identify primary tephras (tephras that have not suffered any reworking process) is detailed inspection at the microscope [57]: Non-reworked volcanic sediments contain characteristic glass shards, often vesicular pumice fragments preserved in sediments, see Fig 4. We used the presence or absence of these shards as attested by the experts to confirm or deny the reality of all the candidate tephras and cryptotephras detected by our method. See [36] for a detailed characterization of the shards present in our cores, including high resolution SEM images.

## Results

**Fig 5:**
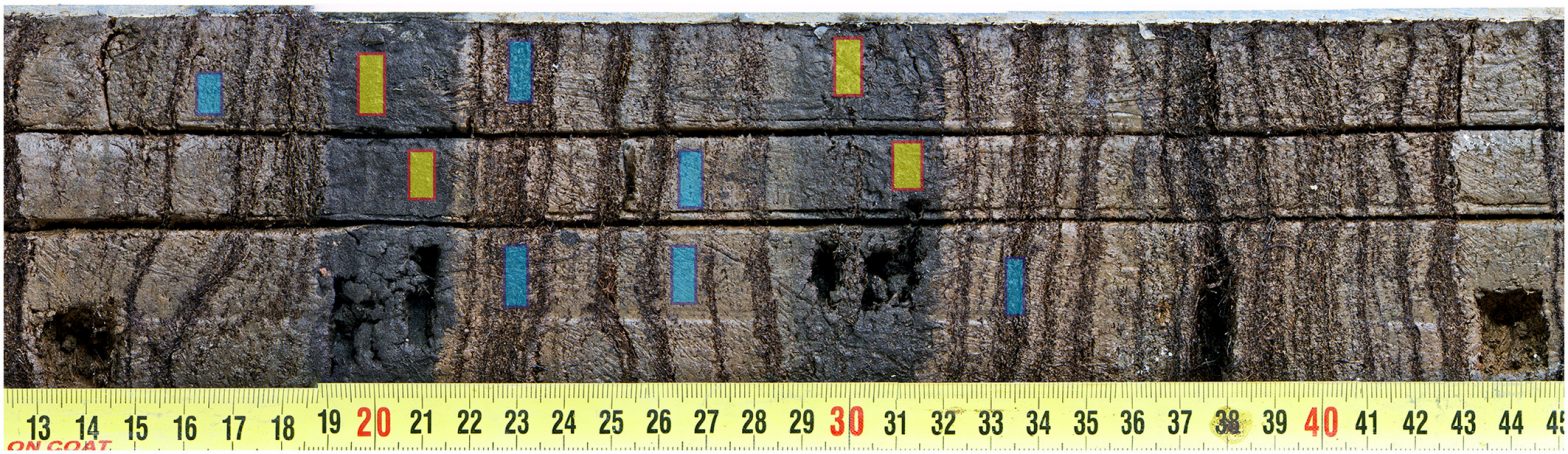
Sediment core profile ES12-0302. The scars from destructive analytical methods are clearly visible. We extracted our training dataset from this core, taking advantage of the two clearly visible tephra layers. The yellow boxes show areas used to take positive samples, the blue boxes the negative samples.

We used the sediment core ES12-0302 from Lake Escondido as our source for the training and validation samples. We chose this core because it includes clearly visible tephra layers alternating with a varied succession of organic and silty clay layers. This sediment core had been previously analyzed with destructive procedures, including geochemical characterization of the tephras [36]. The scars of the previous analyses are clearly visible, see Fig 5). The lithological description of this core highlighted the presence of two 2 cm thick tephras. We extracted 19766 pixel samples of tephra and 40299 pixel samples containing other sediment types (“non-tephra”), including organic substances such as moss and mineral substances such as clay. Then we split the dataset in two to get our training and validation sets.

After testing several configurations, we opted for a 20 × 35 hexagonal SOM. The U-matrix resulting from the unsupervised learning process is shown in Fig 3, together with the confidence matrices computed by supervised classification of both classes. A noticeable “hot wall” can be seen in the U-matrix that clearly matches the “cliffs” acting as class boundaries in each confidence matrix, hinting at good discrimination capabilities for these classes.

We then classified the validation set, and computed and overall accuracy of 98.28% with *κ* 96.13%. These are significantly good values giving the green light to the selected SOM configuration and its training. Thus we proceeded to analyze with it the hyperspectral image data of all the sediment cores.

### Sediment core ES12-0301

**Fig 6:**
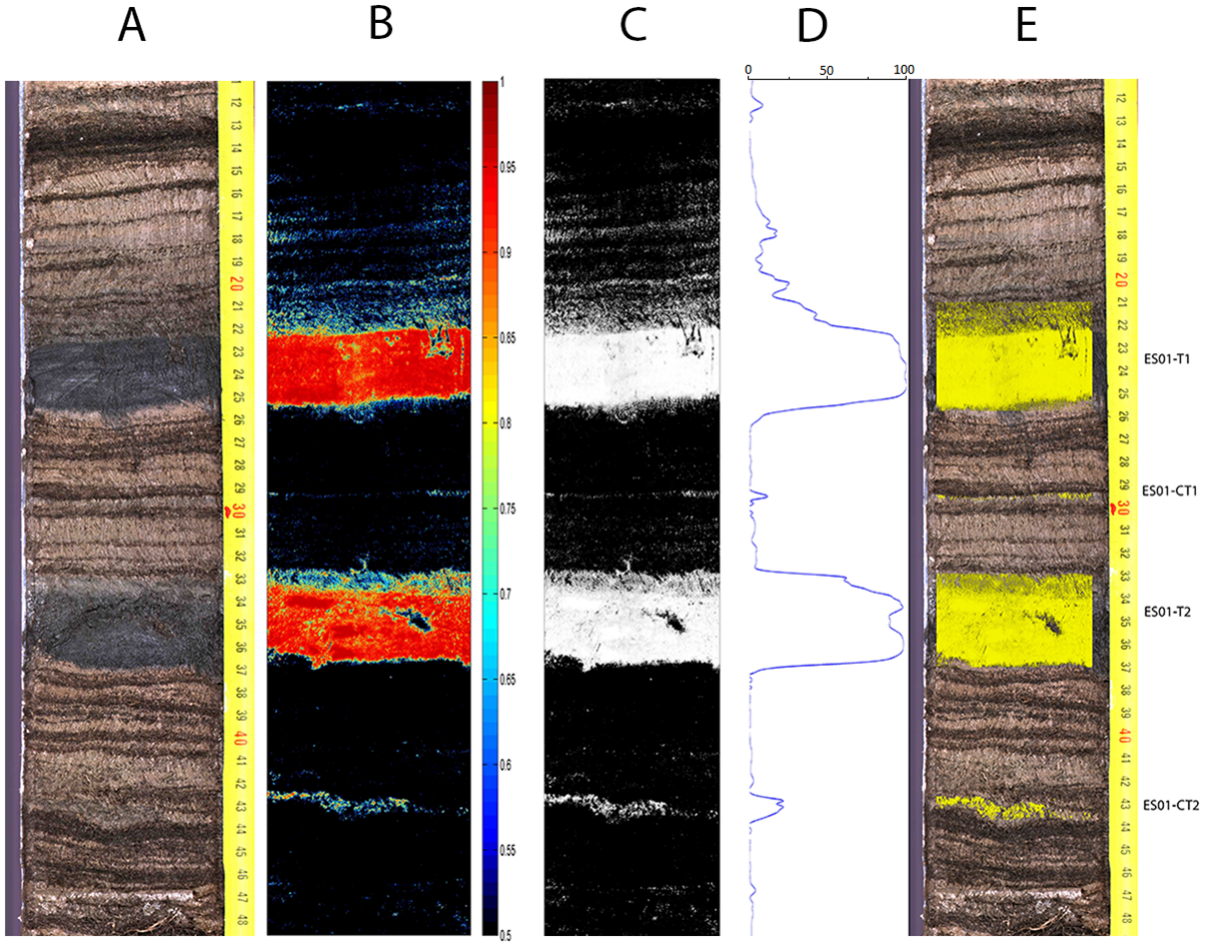
Sediment core ES12-0301 results. A: Referenced color photograph of the core; B: Color coded tephra confidence levels above 0.5; C: Thresholded (0.5) tephra-suspect pixel mask; D: Tephra profile obtained by horizontal projection of the thresholded and morphologically filtered tephra-suspect binary mask; E: Detected tephra layers.

Fig 6 shows the results of tephra detection for a section of a core from the same lake as the one used for training the SOM, side by side with a referenced color photograph of the core. A confidence threshold of 0.5 was used for the detection of tephra-suspect pixels. Confidence values for the tephra class over the detection threshold are first depicted in color scale (the redder the higher), and then the binary mask obtained by thresholding is shown, with all suspect pixels in white, irrespective of their confidence level after thresholding. The confidence level of the tephra-suspect pixels in color scale helps the expert assess the overall confidence level of the system, and also set a different threshold if needed. Fig 6 also shows the tephra profile obtained by horizontal projection of the thresholded and morphologically filtered tephra-suspect binary mask, as explained earlier. The result of the peak detection and selection in the tephra profile is also shown, overlaid on the core photograph: The bright yellow areas indicate detected tephra layers (made of those pixels corresponding to the tephra class above the specified confidence threshold and belonging to a detected tephra profile peak), named on the right side of the photograph. Two thick tephra layers appear, ES01-T1 and ES01-T2 (at 22 –25 cm and 34 –37 cm), together with a cryptotephra layer at 29.5 cm, ES01-CT1, and another one at 43, ES01-CT2. All were confirmed by the presence of volcanic shards, see Fig 4.

### Sediment core LIM08-A2A

**Fig 7:**
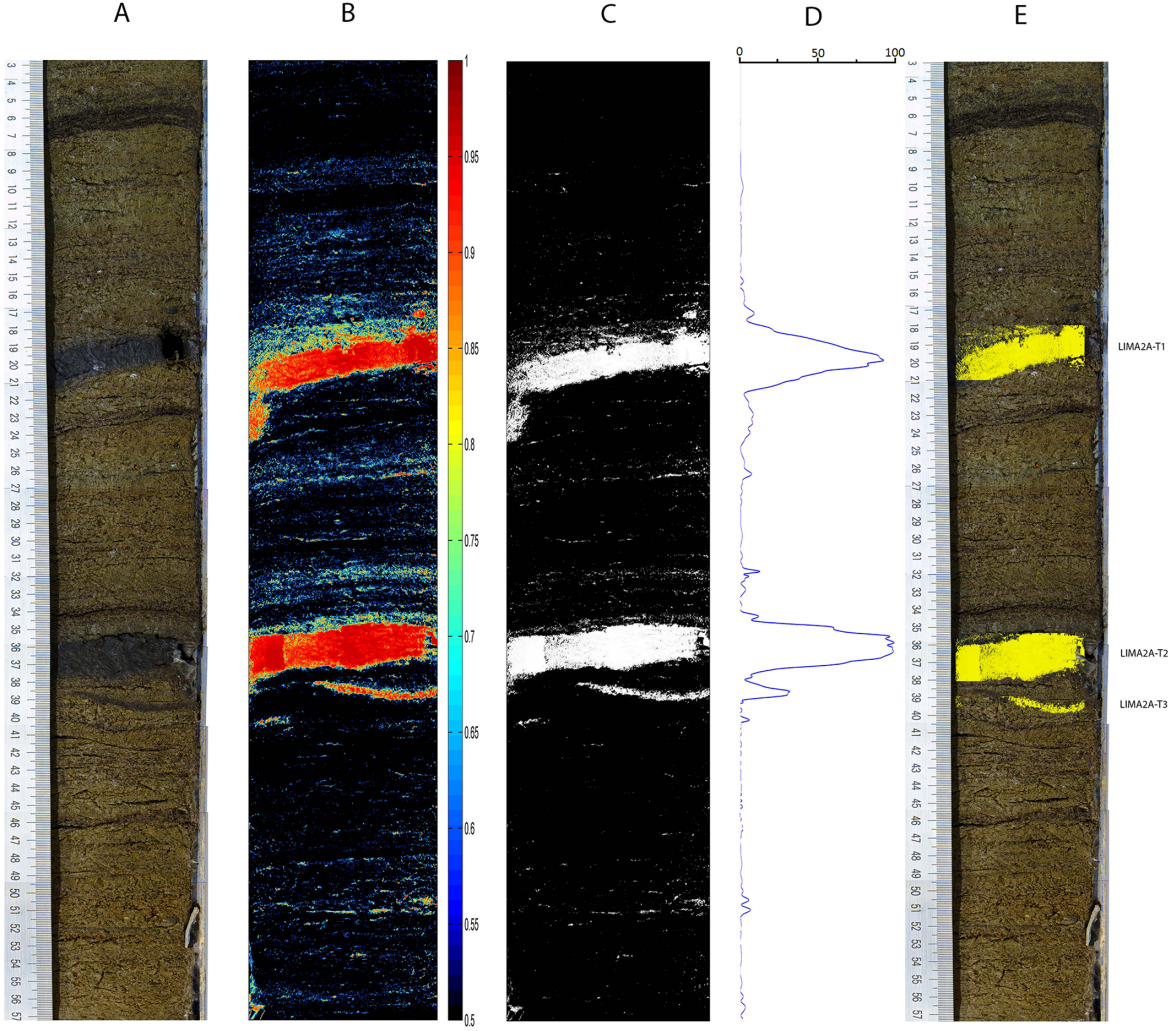
Sediment core LIM08-A2A results. A: Referenced colour photograph of the core; B: Color coded tephra confidence levels above 0.5; C: Thresholded (0.8) tephra-suspect pixel mask; D: Tephra profile obtained by horizontal projection of the thresholded and morphologically filtered tephra-suspect binary mask; E: Detected tephra layers.

The results with core LIM08-A2A, from the Limnopolar lake, are shown in Fig 7. Two thick tephra layers can be readily appreciated in the photograph [42], and were correctly detected. A small tephra was also detected, close to one of the thick tephra layers, and validated by the presence of shards. It is interesting to note the dark layer close to the upper part of the core section in Fig 7, where the method showed high confidence in that it is *not* a tephra, in spite of its appearance. Note also that our SOM was trained with samples from a core from a different lake.

### Sediment core LIM08-F1A

**Fig 8:**
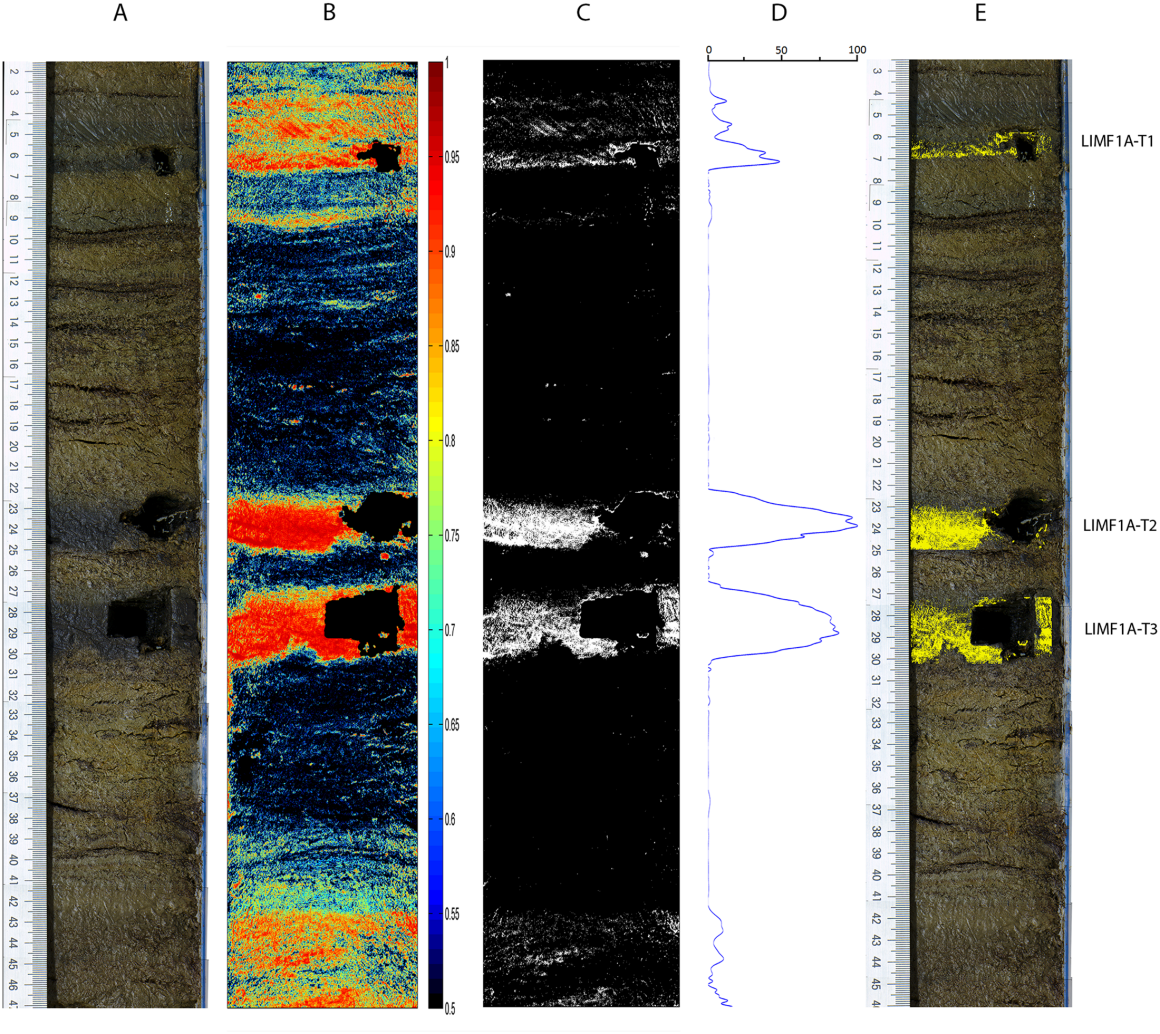
Sediment core LIM08-F1A results. A: Referenced colour photograph of the core; B: Color coded tephra confidence levels above 0.5; C: Thresholded (0.9) tephra-suspect pixel mask; D: Tephra profile obtained by horizontal projection of the thresholded and morphologically filtered tephra-suspect binary mask; E: Detected tephra layers.

This core, also from Limnopolar lake, presented some additional challenges, see Fig 8. Significant areas of the core show reworked volcanic particles which however were not able to trick the method. Areas of reworked particles appear in the color chart of tephra confidence with a characteristic weakness or lack of spatial integrity, hinting the operator towards a higher confidence threshold for this core. In this way three tephra layers were detected, and confirmed by the presence of shards. The impact of previous destructive analytic methods can also be clearly seen.

### Sediment core CN12-0301G

**Fig 9:**
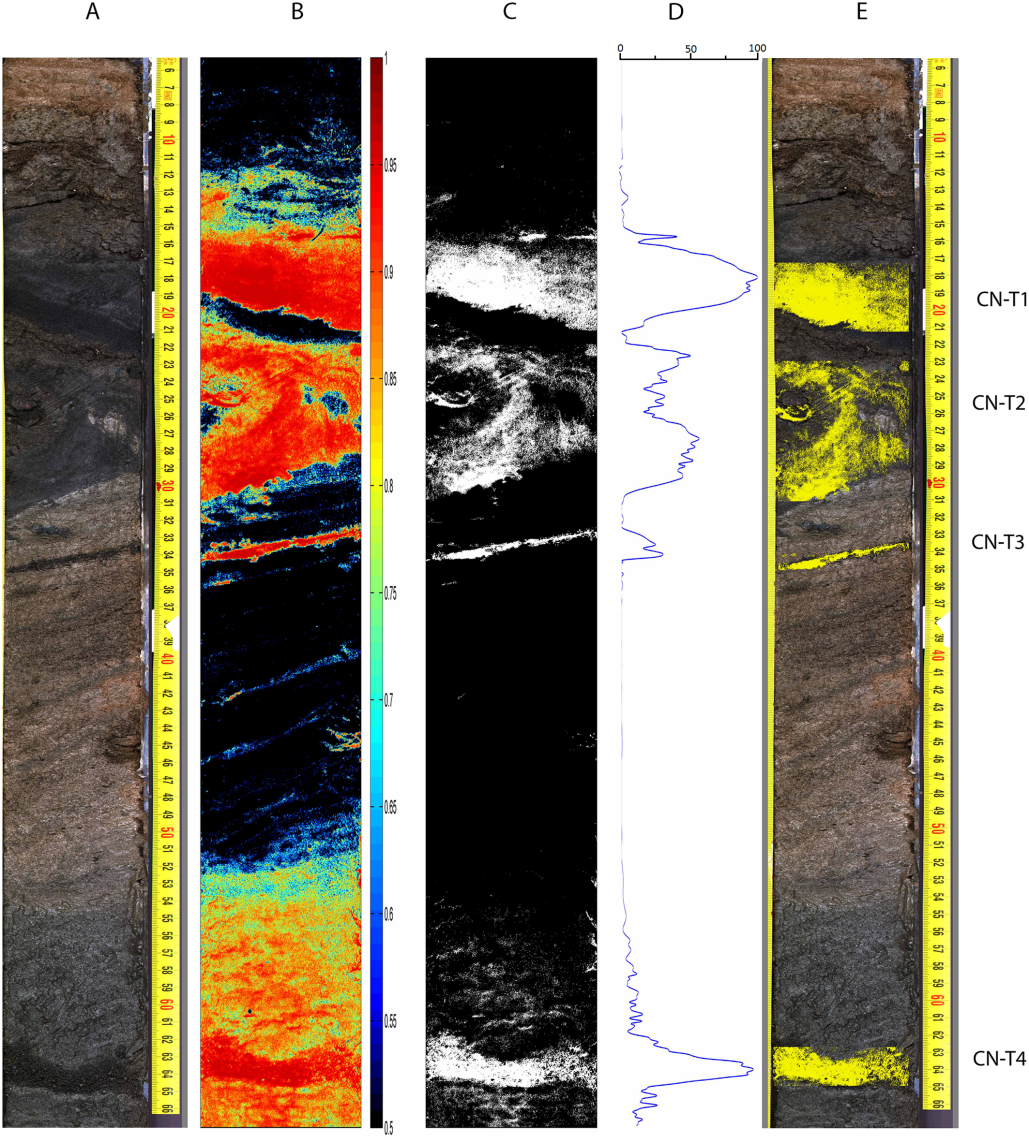
Sediment core CN12-0301G results. A: Referenced colour photograph of the core; B: Color coded tephra confidence levels above 0.5; C: Thresholded (0.9) tephra-suspect pixel mask; D: Tephra profile obtained by horizontal projection of the thresholded and morphologically filtered tephra-suspect binary mask; E: Detected tephra layers.

The core from Cerro Negro lake was a definite challenge, due to its peculiar tephra arrangement, see Fig 9. The region from 17 to 37 cm shows strata of volcanic sediments that have lost their horizontal structure, most likely due to some post-sedimentary process (e.g. slumping). The hyperspectral image combined with the SOM and spatial analysis, however, showed its potential to deal also with these modified tephra layers. In spite of the spatial arrangement deviated from the typical horizontal stratification, and the significant presence of reworked sediments, the detection of tephra suspects and subsequent profiling was able to detect and clearly draw the four tephra layers in the image, once more validated by identification of shards.

### Sediment core ES12-0302

**Fig 10:**
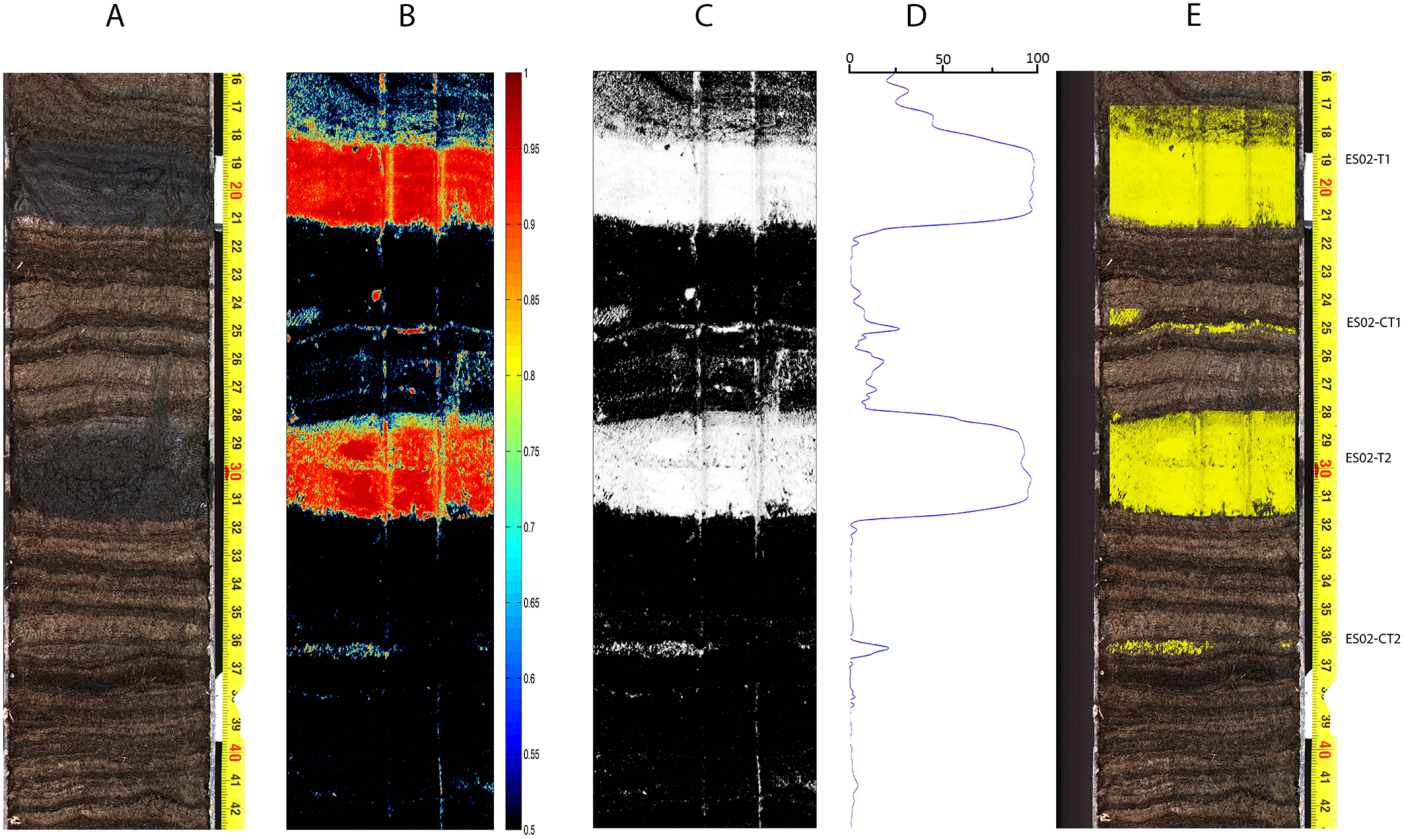
Sediment core ES12-0302 results. A: Referenced colour photograph of the core; B: Color coded tephra confidence levels above 0.5; C: Thresholded (0.5) tephra-suspect pixel mask; D: Tephra profile obtained by horizontal projection of the thresholded and morphologically filtered tephra-suspect binary mask; E: Detected tephra layers.

Finally, Fig 10 shows the results obtained with the sediment core used for training the SOM. As expected, the two big tephra layers from which we extracted the tephra training samples were clearly detected. But not so expectedly, two additional much thinner tephra layers were also detected, ES02-CT1 at 25 cm and ES02-CT2 at 36 cm, and subsequently validated by identification of shards. Note that the artifacts that can be seen in the processed images come from the impact on the core of the destructive analysis carried on it before our hyperspectral scanning of the core, see Fig 5 for the actual state of the core at the time of scanning.

## Discussion

The proposed method has shown promising results. It showed significant consistency when dealing with different sediment cores than those used for training the system, including even different lakes. The detection of thick tephra layers evident to the naked eye was consistent and robust, even in the presence of dark layers that at first sight to the non expert could resemble tephra layers, but which are in fact of a non-volcanic nature. In this respect we could say that the system has effectively learned to replace the expert eye. However, this, even if it may prove useful, is not the primary target of the method. The detection of tephra layers readily noticeable to the expert eye is not a major problem. It is the detection of the other tephra layers, the ones not readily apparent to the expert eye, either due to their slight thickness or to the presence of confusing tephra-like strata in the core, that is in need of fast, reliable and non-invasive techniques. In this respect the proposed method has also shown promising results, being able to detect even some thin tephra layers or cryptotephras that had been skipped by other analytic procedures performed earlier on the cores [36, 42]. Table 2 summarizes the tephra layers detected in the core sections analyzed.

**Table 2:** Tephra layers detected by the proposed method.

**Core**	**Tephra**	**Location**
ES12-0301	ES01-T1	22–25 cm
	ES01-CT1	29.5 cm
	ES01-T2	34–37 cm
	ES01-CT2	43–43.5 cm
ES12-0302	ES02-T1	17.5–21.5 cm
	ES02-CT1	24.5–25 cm
	ES02-T2	28–31.5 cm
	ES02-CT2	36–36.5 cm
LIM08-A2A	LIMA2A-T1	18–21 cm
	LIMA2A-T2	35.5–38 cm
	LIMA2A-T3	39–39.5 cm
LIM08-F1A	LIMF1A-T1	6–7 cm
	LIMF1A-T2	22.5–25 cm
	LIMF1A-T3	27–30 cm
CN12-0301G	CN-T1	17–21 cm
	CN-T2	23–31 cm
	CN-T3	33.5–35 cm
	CN-T4	62.5–65 cm

We cannot warrant that the proposed method has been able to detect every cryptotephra present in the core sections analyzed, but all previously known tephras and cryptotephras in these sections were detected by the method, and even some cryptotephras that were not previously known. This is sufficient proof of the usefulness of the method, which comes at zero impact on the cores, and very little analytical effort. Other techniques, such as magnetic susceptibility, X-ray fluorescence, or shard concentration profiles [8], could be used to compare the results. We measured magnetic susceptibility data using a Bartington point sensor (MS2E) mounted on a MSCL Geotek system, from the Geological Survey of Spain (IGME). However, this sensor has a diameter of 1 cm, every measurement integrates the magnetic susceptibility of 0.5 cm around the sensor tip, and only one measurement was obtained for every centimeter. Our magnetic susceptibility record is thus significantly smoothed, hindering the detection of cryptotephras. In core ES12-0302 it detected only the two main thick tephras, in contrast to the hyperspectral technique. Shard concentration profiles would provide a reliable, if costly, ground truth for validation, recommended for future work. However, note that tephra shard counts necessarily come from a narrow section of the cores, while the tephra profiles obtained with our method take into account lateral variations across the whole section of the core. This introduces an additional source of variation preventing direct comparison with shard count profiles. The same can be said with respect to X-ray fluorescence data, which we intend to obtain in the future.

With the proposed method the cores do not need to go through any thorough preparation procedures, and no singularly expensive equipment is required. The analytic stage is fast and rather straightforward, even if requiring some degree of human intervention for best results in cores with significant presence of reworked particles. But this is dealt with by means of an interactive graphical interface allowing real-time inspection of the effects of the tuning, whose free parameters have an straightforward effect on the results that can be readily understood upon operation, not requiring previous expertise in machine learning techniques.

Moreover, the interaction also allows the expert to target different detection scenarios, depending on the purpose, and therefore either aim for a higher likelihood of false detections, if not missing any potential tephras is important, or rather aim for a very robust detection with very marginal false detection likelihood, for instance if costly analytical procedures, in terms of resources, time or impact on the sample, will follow the hyperspectral analysis. And all the different scenarios can be performed on a unique dataset, the hyperspectral image, that can be stored once and retrieved as many times as desired, to repeat the analysis with different target scenarios or to confirm or contrast later results from other analytical sources.

The tunable thresholds for the confidence level in the detection of tephra pixels and for the selection of peaks in the detection of tephra layers is useful for studying reworking of tephra. Different settings will bias the results towards stricter or looser requirements regarding the spatial arrangement and the concentration of the tephra layers. Different threshold settings are able to include or exclude reworked tephra (e.g. see Fig 9), and also to increase the sensitivity in cryptotephra detection at the cost of a higher risk of false positives. The realtime feedback of the final detection while adjusting the thresholds combined with the expertise of the operator provides useful insight about the reliability, significance and ultimate interpretation of the results.

The most labor-intensive phase of the process is the selection of the training and validation sets for the SOM. Unequivocal tephra and non-tephra samples (pixels) have to be found in at least one of the cores in sufficient amount. But, as we have just seen, the training with samples from a core can be successfully used for analyzing other cores, not even from the same basin. So we are quite confident that a well trained SOM can be used for a wide range of cores, even from different field campaigns, as long as the scanning conditions (hyperspectral system, lamp, resolution) are kept equal and the array of significant substances in the new cores was also well represented in the core used for training. And if it is otherwise, the cost of retraining the system does not seem a high price in view of the results presented here.

In this regard, note that the geochemical composition of the tephra layers detected has allowed to define their source in Deception Island, distant 25–30 km from Byers Peninsula. As stated in [36], “the compositional range of each tephra layer is relatively uniform, with small standard deviations. There is no obvious contribution from multiple volcanic sources. Macrotephra thicknesses and textural/compositional trends are regionally consistent between all cores from Byers Peninsula.”

The detection of tephras of different compositions would mainly depend on the training set and on the spectral differences between the different tephra types. If the intraclass differences between different tephra types as projected in the SOM manifold when trained with samples of just one tephra type were higher than the interclass differences between the tephra and the non-tephra classes, a training set including all types of tephras would be required in order to conform the map to a manifold where intraclass differences were lower than interclass differences. Note that preprocessing methods have been devised for hyperspectral imagery that are able to reduce intraclass variances while preserving or even increasing interclass variance [58, 59], and these could be used as a step previous to classification if deemed necessary.

Variability of tephra composition is not the only potential source for relative increases of intraclass variability. Relatively significant intraclass differences in the non-tephra class could overwhelm diminished interclass (tephra:non-tephra) differences in some cases, for instance in marine sediments. Depending on the distance to the sources, basin topography, marine currents, and prevailing winds, tephra layers can be subjected to reworking and/or depositional processes which may interfere in the geochemical signal of the tephras, approximating them to those of the host sediments. Therefore, it may be harder to distinguish tephra in marine sediments. By contrast, in other records such as ice cores, where fallout is direct and (almost) no reworking exists, interclass differences are expected to be very high. Plenty enough to reliably overpower the expected increase in intraclass variability in ice cores from the Antarctic continent due to the wide range of volcanic sources [60].

The need of a training set may be a problem when working with cores not containing any visible horizons from which to extract positive samples. This can be addressed either by testing training data from cores from other areas where visible horizons do exist, or by first identifying one or several cryptotephras in the core by means of shard counts or other suitable technique, prior to the selection of training samples and just to provide enough training samples. Alternatively, note that the U-matrix does not need any labeled samples: It just indicates the presence of separable clusters in feature (hyperspectral) space. Unlabeled random samples could be used to train the SOM, and any hot ridges in the resulting U-matrix could be used to define cluster boundaries. The lack of labeled training samples would prevent the availability of confidence matrices, but a blind detection could be subsequently performed in the core by just selecting all pixels mapped to a given region of the U-matrix. Which region of the U-matrix, if any, could correspond to tephra pixels would then have to be determined by checking the presence or absence of volcanic shards in the corresponding regions of the core.

## Conclusions

The purpose of this paper was to find a reliable, fast, and non-destructive method to detect tephra layers, including those hardly identifiable by the naked eye. We were especially interested in detecting tephra layers in sediment cores collected from Antarctic lakes in the ice-free area of the Byers Peninsula. Five sediment cores from three different lakes (Escondido, Limnopolar, and Cerro Negro) were analyzed in previous studies using traditional methods [36, 42] in order to determine the existence of volcanic ashes and characterize them. The proposed method was able to detect all the tephra layers already known, together with several cryptotephras not previously known. The identification of cryptotephras allows to fill the gaps in the history of volcanic activity of Deception Island during the Holocene. This knowledge is crucial for many paleoenvironmental and paleoclimatic studies in the Antarctic Peninsula region.

Hyperspectral scanning was recently used in lake sediment records to infer past environmental and climatic changes [25]. Here, coupled with machine learning and image processing techniques, we have shown its applicability to detect also past volcanic eruptions preserved in the lacustrine sedimentary record. The potential for paleoclimatic and paleoenvironmental studies in other type of records deserves further study. Within Antarctic research, tephra layers are isochrones widely used by the ice core research community to validate the absolute chronology established by the counting of annual ice layers [61, 62]. However, volcanic aerosols in the Antarctic ice sheet come from a wide source of polar, mid-latitude and tropical volcanoes, and therefore their geochemical signals are different [60]. Hyperspectral scanning of the ice cores could be used to automatically detect cryptotephras as well as to group different geochemical volcanic signals attributed to the same source in a rapid and efficient way. This approach may be also applied to marine sediment cores collected from the waters surrounding the Antarctic continent where visible tephra layers are present [63, 64], though no accurate studies have been conducted to infer the tephrochronological history in the area.

As an added value, the hyperspectral images of the cores, containing the reflectance spectral signatures of every pixel at high or very high spatial resolution, provide also a maintenance-free digital record susceptible of future analyses using different hyperspectral analytic tools, be it for tephra detection or for other purposes of interest in sedimentology: The reflectance spectra may be a useful source of information about past environmental and climatic events not necessarily associated to volcanic activity. Even long after the cores themselves have been lost to destructive analysis. Or to time: dust to dust, ashes to ashes.
